# Editorial: Incorporating technology for teaching vocabulary to second language learners

**DOI:** 10.3389/fpsyg.2022.1081901

**Published:** 2023-01-20

**Authors:** Ehsan Rassaei

**Affiliations:** Majan University College, Muscat, Oman

**Keywords:** vocabulary, technology, call, L2, smartphones, language learning

Since learning vocabulary plays an essential role in second language (L2) acquisition, a lot of attention has been paid to developing techniques as well as investigating factors that lead to and promote L2 vocabulary learning (e.g., Nassaji and Tian, 2010; Huang and Lin, 2014; Sun, 2017; Rassaei, 2018; Nguyen and Boers, 2019). The bulk of the extant research on L2 vocabulary has been devoted to investigating techniques that are beneficial to L2 vocabulary leaning. These studies highlighted the importance of several factors which should be considered in designing L2 vocabulary learning tasks. One such factor is the level of L2 learners' engagement with the learning task. According to the involvement load hypothesis (Hulstijn and Laufer, 2001), the level of learners' cognitive involvement with the vocabulary learning task, which is determined by three factors namely search, choice, and evaluation, is essential for vocabulary learning. An implication of this hypothesis is that vocabulary learning tasks that are more cognitively engaging are more effective for L2 vocabulary learning. Further research also indicated the benefits of productive vocabulary learning activities that engage learners in recycling new words productively while performing learning tasks (e.g., Laufer, 2006; Keating, 2008; Min, 2008; Rassaei, 2017). These studies in general suggest that activities that result in more elaborative processing lead to more fruitful results.

Meanwhile, as L2 acquisition researchers have become increasingly more interested in applying different forms of technology in language learning, interest in incorporating technology to teach vocabulary has been also growing in recent years. An increasing number of researchers have become interested in investigating the effects of different forms of technology in vocabulary learning (e.g., Lin and Yu, 2017; Rassaei, 2020; Teng and Zhang, 2021; Lo, 2022; Muñoz et al., 2022). Several features of Web-2 technology such as sharing information and interaction in real time which promote collaborative learning made various forms of technology appealing to SLA researchers. Moreover, the availability of portable devices such as smartphones which provided further affordances for language learning has sparked an increasing number of studies with regard to vocabulary learning (e.g., Lin and Yu, 2017; Xu and Peng, 2017). Various technology tools such as computer applications, virtual spaces and other digital resources have made collaborative language learning easier and more successful among L2 learners (Gánem-Gutiérrez, 2018).

In line with the current interest in applying various forms of technology for vocabulary learning and instruction, the present article Research Topic aimed to collect and present a number of studies that investigated how technology can be used to teach vocabulary to L2 learners. As the first study in this Research Topic, Soleimani et al. investigated how smartphones can provide assistance for vocabulary learning. In their study, a group of EFL learners read a number of short texts that included some unfamiliar vocabulary items. Another group of participants read the same texts and listened to the audio file of the same texts through their smartphones. The results revealed the value of smartphones in enhancing L2 vocabulary knowledge. The findings also indicated the benefits of smartphones in increasing learners' vocabulary learning self-efficacy. The effectiveness of smartphones for L2 vocabulary learning has also been investigated by other studies in the present article Research Topic. Polakova and Kimova examined the effects of a mobile application that provided several affordances for L2 learners including translation, text to speech and pronunciation functions. The results revealed that those students who used the application improved in vocabulary learning and experienced higher motivation and satisfaction for vocabulary learning.

In a longitudinal study, Lei et al. examined the effects of mobile-assisted vocabulary learning on EFL learners' attitudes toward vocabulary learning and their self-regulation. The results revealed the positive effects of mobile-assisted vocabulary learning on learners' vocabulary learning attitudes and their self-regulation scores. These three studies in the present Research Topic provided evidence for the value and effectiveness of smartphones in enhancing L2 learners' vocabulary knowledge as well as promoting L2 learners' attitudes toward vocabulary learning.

The affordances provided by smartphones for L2 vocabulary learning have also been examined by two other papers in the present Research Topic. Xodabande et al. investigated how digital flashcards, installed on the participants' smartphones, can be used for teaching academic words to university students. The results indicated that the participants who used digital flashcards outperformed the group that used traditional paper flashcards as well as the control group. Rahmani et al. also investigated how a digital flashcard application installed on smartphones could enhance EFL learners' vocabulary knowledge. The results were promising as those students who used the application outperformed the control group that was exposed to regular language learning activities in the absence of the digital flashcard. The results of the studies published in the present Research Topic and reviewed above, point to the benefits of smartphones and the relevant applications installed on them for teaching vocabulary to L2 learners.

Turning to other studies published in this Research Topic, Jiang et al. investigated the effects of automatic speech recognition (ASR) technology on vocabulary learning in a tertiary flipped setting. The results indicated that the group who incorporated ASR technology into their classroom activities outperformed the control group that did not use ASR during the treatment session in terms of vocabulary learning.

Two studies in the present Research Topic adopted a research synthesis approach to investigate the use of technology for teaching vocabulary to L2 learners. Wei and Fan reviewed the studies that investigated the effects of different forms of on-screen texts such as subtitles and captions on L2 vocabulary learning. The authors reported L2 captions to be more effective than (L1) subtitles. The authors also reported that some individual learner factors can also moderate the effectiveness of on-screen texts for vocabulary learning. Lei and Reynolds also synthesized research on learning L2 English vocabulary from word cards based on 32 primary studies. The authors reported a larger effect size for studies that investigated paper word cards than digital word cards and also for the ready-made word cards compared with self-constructed word cards. One reason for why learners benefited more from paper word cards compared to digital word cards can be attributed to learners' familiarity with paper word cards and their lack of experience to work with digital word cards.

In another study, Reynolds et al. investigated the impact of listening to audio-recorded lectures on incidental vocabulary learning. The learner participant of the study listened to the audio-recorded lectures provided *via* Google Drive for about 14 weeks. The findings indicated the benefits of listening to the lectures for incidental vocabulary learning. The student also indicated positive attitudes toward listening to the lectures for vocabulary learning. Finally, two studies in this Research Topic investigated the affordances provided by corpora for L2 vocabulary learning. Bao and Liu examined how corpora can be used to compare the differences in the use of lexical bundles between Chinese and American students writing their Ph.D. dissertations' abstracts. The findings indicated major variations in the use of lexical bundles in both groups. The results provided evidence for the importance of using corpora for identifying lexical bundles which pose difficulty for language learners. Finally, Zhang explored the use of online corpora to enhance learners' morphological knowledge and L2 vocabulary learning. The findings indicated the conducing effects of using corpora in language classrooms to enhance vocabulary learning.

In sum, the articles published in this Research Topic referred the affordances that technology can provide for L2 vocabulary learning. In particular, these studies indicated the benefits of mobile-assisted language learning in L2 vocabulary learning. Further technology affordances for L2 vocabulary learning as indicated in the present Research Topic include on-screen texts, digital flashcards, audio-recorded lectures, digital corpora and speech recognition technology. These studies provided evidence for the values and the effectiveness of different forms of technology. Moreover, the studies indicate a variety of technology tools that can be utilized in various contexts depending on factors such as learning and teaching styles as well as their availability. In addition to the evidence provided by studies in the present Research Topic regarding the usefulness of the technology tools in enhancing L2 collaborative vocabulary learning, one implication which can be inferred from these studies is the diversity and variety of the technology tools that can be adapted by language teachers to suit specific purposes in specific contexts.

## Author contributions

The author confirms being the sole contributor of this work and has approved it for publication.
